# Dr. Lucien Howe

**Published:** 1887-12

**Authors:** 


### *DR. LUCIEN HOWE.*

The many patients and friends of Dr. Howe will learn with regret that he has arranged for a prolonged absence from the city.

The object which he has in view is, however, one so praiseworthy, and the sacrifices involved in its fulfillment so great, that the profession will join with us in the hope that his researches will result in the advancement of medical knowledge, and renewed honors to himself.

The last census shows that, during the decade ending in 1880, blindness has increased four times as fast as the population. At the last meetings of the New York Medical Society and the American Opthalmological Society, the subject was discussed, and it was shown that eye diseases in this country are largely due to contagion.

Both of these societies appointed committees to investigate and discover the best remedy for this growing evil, and Dr. Howe was honored by being made chairman of both committees.

It is for the furtherance of these investigations that Dr. Howe has determined on relinquishing, for a time, his lucrative practice, and exiling himself to Egypt and other eastern countries, where contagious eye diseases are peculiarly prevalent, there to pursue the study of these affections in the light of more recent bacteriological knowledge.

As he has been officially accredited by the U. S. Government, he will receive every possible assistance in his studies from our representatives abroad, and, knowing the energy, ability for work, and scientific attainments of our quondam editorial colleague, we feel sure that important results will be obtained.

Dr. Howe proposes to sail from New York December 3d, and it is hoped that he will be able to return early next summer.

